# Research on the role and mechanisms of Cystatin 6 in disease pathosis and development

**DOI:** 10.3389/fmolb.2026.1768124

**Published:** 2026-03-13

**Authors:** Yunhua Li, Xuehong Long, Gangwen Chen, Yongkang Wu, Honghua Wen, Chunrong Tang, Yi Liu

**Affiliations:** 1 Department of Radiology, West China Hospital Sichuan University Jintang Hospital·Jintang First People’s Hospital, Chengdu, Sichuan, China; 2 Hospital Infection Control Department, West China Hospital Sichuan University Jintang Hospital·Jintang First People’s Hospital, Chengdu, Sichuan, China; 3 Department of Laboratory Medicine, West China Hospital, Sichuan University, Chengdu, Sichuan, China

**Keywords:** biomarker, Cystatin 6 (Cystatin E/M), non tumor diseases, protease inhibitor, therapeutic target, tumor promotion, tumor suppression

## Abstract

The Cystatin 6 (CST6 [also known as Cystatin E/M]) is a cysteine protease inhibitor that exhibits a dual role in various types of tumors, acting as either a suppressor or promoter. In non-oncological contexts, CST6 maintains skin barrier homeostasis, mediates preeclampsia pathosis, and is associated with conditions such as pulmonary fibrosis. Diagnostic approaches involving CST6 include liquid biopsy and tissue analysis, while therapeutic strategies involve epigenetic activation and recombinant protein administration, among others. Overall, as a key variable driving multisystem pathologies through protease-substrate imbalance, CST6 represents a molecular target for precision diagnostics and therapeutics across diseases, necessitating in-depth investigation into its functions. Based on the existing research, this review summarizes the fundamental theory of CST6 in disease, considers its roles in both oncological and non-oncological conditions, and proposes future research directions.

## 1 Introduction

CST6 is a key regulatory protease inhibitor. The dysregulation of CST6 expression or function drives the pathosis of multisystem diseases. CST6 serves not only as a significant cross-disease diagnostic biomarker—particularly in liquid biopsy—and has been used in early renal disease detection, cancer classification, and prognosis prediction. CST6 also represents a highly promising therapeutic target owing to its involvement in core pathological mechanisms, such as the inhibition of osteoclast differentiation, maintenance of skin barrier integrity, and modulation of the immune microenvironment. Future efforts to elucidate the mechanisms underlying its functional paradox, develop targeted therapeutics, and explore precise combination treatment strategies may enable CST6 to play a transformative role in the precision medicine of various diseases, including cancer, dermatological disorders, and kidney conditions.

## 2 Molecular biological characteristics of CST6

Cystatins, encoded by the CST family, constitute a superfamily of cysteine protease inhibitors that are widely involved in diverse biological functions (Xu et al., 2025). Cystatins are universally present in animals, plants, and microorganisms (Shamsi and Bano, 2017; Turk et al., 2008). Based on structure, localization, and function, cystatins are classified into three major types: Type 1 (Cystatin A/B), primarily intracellular and non-glycosylated single-chain proteins (approximately 100 amino acids, 11 kDa) that lack disulfide bonds and function as monomers to regulate intracellular protease activity; Type 2 (Cystatin C, D, E/M, F, G, S, SN, SA) are mainly extracellular single-chain polypeptides (approximately 120 amino acids, 13 kDa), typically stabilized by two disulfide bonds and subject to glycosylation or phosphorylation, and are involved in immune defense and regulation of cancer biology; and Type 3 (HMWK/LMWK), intravascular proteins distributed in plasma and tissue fluids, which are multi-domain proteins (containing four cystatin domains) that function both as carriers of coagulation factors and protease inhibitors (Abrahamson et al., 2003; Ni et al., 1997). Their main target enzymes include cathepsins, papain, and calpains.

### 2.1 Protein structure of CST6

The CST6 is located on human chromosome 11 and contains the tic instructions required for the production of the Cystatin-6 protein. The DNA sequence of the CST6 is transcribed into mRNA and subsequently translated into the protein which is referred to as CST6. The core structure of the protein consists of five β-strands and one α-helix. Through three key functional elements—the N-terminal glycine-valine-glycine flexible loop, the β1 strand, and the β2 strand—this protein precisely binds to the active site of cysteine proteases, blocking substrate access. Structural stability is ensured by two disulfide bonds, which maintain overall conformational rigidity and confer resistance to proteolytic degradation. The CST6 protein exists in both non-glycosylated (∼14 kDa) and glycosylated (∼17 kDa) forms. Glycosylation occurs at the Asn119 residue; however, the inhibitory properties of CST6 are independent of its glycosylation status. Complex glycosylation may influence protein secretion and cellular localization (Lunde et al., 2017). Based on the crystal structure of human Cystatin D, a three-dimensional model of human CST6 has been constructed, confirming that its overall folding characteristics are highly consistent with those of Cystatin D (Cheng et al., 2006).

### 2.2 Functions of CST6

CST6 is confined to the granular layer in normal human skin, extends to both the granular and spinous layers in psoriatic skin, and is highly expressed in the secretory coils of eccrine sweat glands. Low levels are expressed in nasal tissues. CST6 serves as an efficient substrate for tissue transglutaminase and human stratum corneum extract transglutaminase, functioning as an acyl acceptor but lacking acyl donor activity. In terms of protease inhibition, CST6 exhibits moderate inhibitory activity against Cathepsin B but has no inhibitory effect on Cathepsin C (Zeeuwen et al., 2001; Zhou et al., 2020). During terminal differentiation of the epidermis, CST6 participates in cornified envelope formation as a transglutaminase substrate and is a key regulatory factor in skin barrier function (Zeeuwen et al., 2001). As an epithelium-specific protease inhibitor, CST6 plays important roles in epidermal differentiation and tumor suppression, regulating the activity of legumain, Cathepsin L, Cathepsin V, and transglutaminase 3, thereby contributing to epidermal differentiation. It serves as a core regulatory axis essential for maintaining the integrity of the epidermis, hair follicles, and corneal epithelium. In mouse models, CST6 dysregulation can lead to neonatal lethality, cicatricial alopecia, and keratitis (Zeeuwen et al., 2010). CST6 ameliorates bone loss in multiple myeloma (MM) or ovariectomy (OVX) models by increasing estrogen receptor expression and elevating estrogen concentrations in osteoclast precursors, thereby inhibiting their maturation (Gai et al., 2024). CST6 suppresses the proliferation, colony formation, migration, and invasion of breast cancer cells. Soluble CST6 derived from cancer cells inhibits cancer cell motility. Animal studies showed that ectopic expression of CST6 in cancer cells protected mice from overt osteolytic metastasis and death, whereas knockdown of CST6 significantly enhanced bone metastasis and shortened survival (Jin et al., 2012). Failure of the biological mechanisms controlling protease activity contributes to numerous diseases, such as neuroderation, cardiovascular diseases, osteoporosis, arthritis, and cancer (Turk et al., 2008). Cysteine proteases are closely associated with cancer progression (Hunaiti et al., 2020), and cystatins participate in cancer development and progression by modulating cysteine peptidase activity and proteolysis, influencing tumor growth, invasion, and metastasis (Shamsi and Bano, 2017; Rivenbark and Coleman, 2009).

### 2.3 Mechanisms of CST6 in cellular signaling pathways

#### 2.3.1 Tumor suppressor-related pathway

**FIGURE 1:**
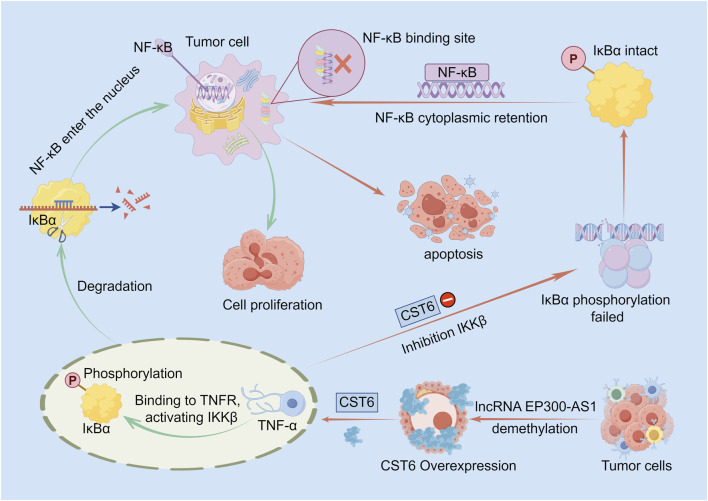
Schematic diagram of the mechanism by which CST6 inhibits tumor cell growth by regulating the NF-κB signaling pathway. In the absence of CST6, TNF-α activates IKKβ, triggering IκBα degradation and subsequent nuclear translocation of NF-κB to promote cell proliferation (green arrows). In contrast, CST6 overexpression—driven by lncRNA EP300-AS1 or promoter demethylation—suppresses IKKβ phosphorylation, thereby stabilizing IκBα, sequestering NF-κB in the cytoplasm, abrogating target gene transcription, and inducing tumor cell apoptosis (red arrows).

CST6 induces apoptosis (both cell autonomous and non cell autonomous) and inhibits tumor cell growth. The specific mechanisms include reducing phosphorylation of IKKβ and IκBα upon tumor necrosis factor (TNF)-α stimulation. Xenograft tumor experiments in nude mice further demonstrated that induced expression of CST6 suppresses tumor growth. Clinical sample analysis revealed a statistically significant negative correlation between CST6 and Cathepsin L expression and a direct relationship between the loss of CST6 and nuclear expression of NF-κB. Thus, the core function of CST6 as a tumor suppressor lies in its regulation of the NF-κB signaling pathway (Soh et al., 2016) (Figure 1).

**FIGURE 2:**
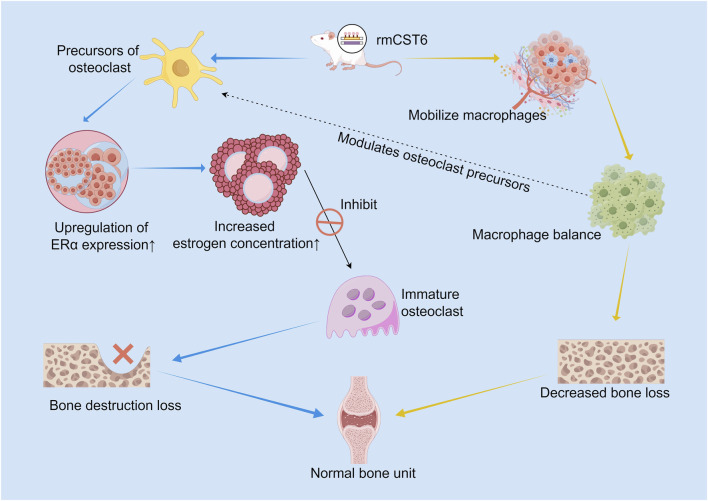
Dual mechanisms of recombinant mouse CST6 (rmCST6) in suppressing osteoclastogenesis and maintaining bone homeostasis. ERα/estrogen signaling (blue arrows).: rmCST6 upregulates ERα expression and elevates estrogen levels in osteoclast precursors, thereby inhibiting osteoclast maturation and alleviating bone destruction. Immunomodulatory pathway (orange arrows).: rmCST6 modulates the balance of bone marrow macrophages, which in turn regulate osteoclast precursors (dashed line), synergistically reducing bone loss. The bone-protective effect of rmCST6 is comparable to that of zoledronic acid, with the unique ability to restore ERα levels in bone tissue.

Targeting Osteoclast Differentiation Pathway: The cysteine protease inhibitor CST6 demonstrates dual therapeutic potential in skeletal diseases. Estrogen receptor alpha (Erα)/Estrogen Axis: In both MM and OVX-induced osteoporosis models, recombinant mouse CST6 (rmCst6) effectively inhibited osteoclast maturation by upregulating ERα expression and increasing estrogen concentration in osteoclast precursors. Its bone loss amelioration effect is comparable to that of zoledronic acid, while uniquely modulating the bone marrow macrophage population and restoring ERα levels in bone tissue (Gai et al., 2024) (Figure 2).

**FIGURE 3:**
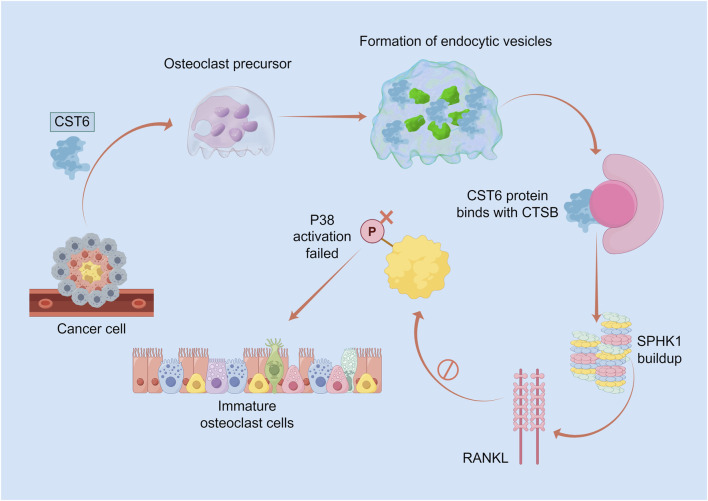
Schematic diagram of the mechanism of the CST6–CTSB–SPHK1 signaling axis in breast cancer bone metastasis. Tumor-derived CST6 (blue) is endocytosed by osteoclast precursors (purple), forming endocytic vesicles (green). Intracellular CST6 binds to and inhibits CTSB (pink) activity, leading to accumulation of its substrate SPHK1. This suppresses RANKL-induced p38 MAPK activation (labeled “P38 activation failure” in the figure), thereby blocking the osteoclast differentiation process and resulting in accumulation of immature osteoclasts.

CST6-CTSB-SPHK1 Signaling Axis: Studies in breast cancer bone metastasis have shown that tumor-derived CST6 enters osteoclasts via endocytosis and inhibits Cathepsin B (CTSB) activity, leading to the accumulation of its substrate, SPHK1. This accumulation blocks the canonical RANKL/RANK/TRAF6/p38/NFκB signaling axis—the central driver of osteoclastogenesis. In this pathway, RANKL binding to RANK recruits TRAF6, initiating sequential activation of p38 MAPK and NFκB, which translocates to the nucleus to induce osteoclast-specific gene transcription. By suppressing RANKL-induced p38 phosphorylation and NFκB activation, accumulated SPHK1 disrupts this cascade, ultimately inhibiting osteoclast differentiation. Consistent with this mechanism, CST6-mimetic peptides and recombinant CST6 proteins significantly suppress tumor-induced osteoclastosis and bone metastasis in preclinical models (Li et al., 2021) (Figure 3).

#### 2.3.2 TBX2-CST6-legumain (LGMN) oncogenic signaling axis

**FIGURE 4:**
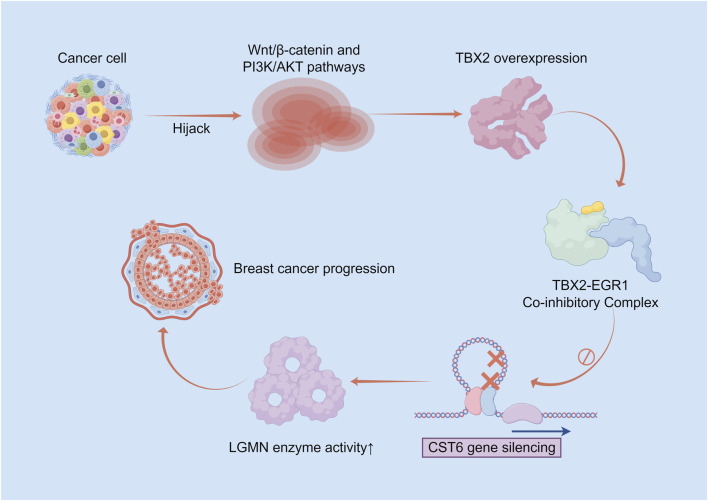
Schematic diagram of the oncogenic mechanism of the TBX2–CST6–LGMN signaling axis in breast cancer progression. Cancer cells hijack developmental signaling pathways (e.g., Wnt/β-catenin and PI3K/AKT), leading to TBX2 overexpression. TBX2 forms a transcriptional corepressor complex with EGR1 to suppress the transcription of the tumor suppressor CST6. Downregulation of CST6 enhances LGMN activity, thereby promoting cancer cell proliferation, survival, and anti-apoptotic capabilities, ultimately driving malignant tumor progression.

The T-box transcription factor TBX2 regulates critical processes during embryonic development, and its reduced levels lead to developmental defects. However, this factor is overexpressed in multiple cancers and exerts potent oncogenic functions, driving cancer cells to evade senescence and death, promoting proliferation, inducing epithelial-mesenchymal transition, facilitating invasion and metastasis, and conferring therapy resistance. TBX2 transcriptionally represses multiple tumor suppressor s—including p21Cip1, p14/p19ARF, PTEN, and CST6—by recruiting repressor complexes. In breast cancer, TBX2 suppresses CST6 expression through cooperative inhibition with EGR1; exogenous expression of CST6 specifically induces apoptosis in TBX2-positive cancer cells via a mechanism dependent on inhibition of the asparaginyl endopeptidase legumain (LGMN). The tumor-suppressive function of CST6 occurs intracellularly. Research has shown an inverse correlation between TBX2 and CST6 in breast cancer, with high TBX2 expression associated with increased metastasis and reduced survival. The oncogenic function of TBX2 can be induced by developmental signaling pathways hijacked by cancer cells (e.g., Wnt/β-catenin, PI3K/AKT). TBX2 overexpression leads to CST6 suppression and LGMN activation, thereby driving cancer cell proliferation and anti-apoptosis, ultimately promoting malignant tumor progression (D’Costa et al., 2014; Paulus et al., 1995) (Figure 4).

#### 2.3.3 Promotion of microenvironment dysregulation

CST6 drives multisystem microenvironment dysregulation through protease imbalance. In preeclampsia, placental hypoxia induces significantly elevated CST6 expression while reducing the target enzyme asparaginyl endopeptidase LGMN, leading to an imbalanced CST6/LGMN ratio in the circulatory system. This imbalance promotes vascular pathology via endothelial dysfunction and placental abnormalities (Botha et al., 2025). In keratolytic winter erythema (keratosis follicularis spinulosa decalvans [KFSD]), a heterozygous missense mutation in the CST6 causes loss of protein function, impairing its ability to inhibit cathepsins (CTSL/CTSV) and stabilize transglutaminases (TGM1/TGM3), resulting in dysregulated terminal epidermal differentiation and abnormal follicular keratinization (Eckl et al., 2021). In *Candida* glabrata (C. glabrata) infections, SNP variation in the 5′UTR region of the CST6 affects azole drug susceptibility, indirectly promoting a fungal adaptive evolution within the host microenvironment and exacerbating drug resistance and virulence microenvironment formation (Guo et al., 2020). The abnormal expression, loss of function, or SNP variation of CST6 disrupts protease-substrate balance, triggering microenvironment homeostasis dysregulation across diverse biological contexts.

#### 2.3.4 Core mechanisms of protease homeostasis regulation

The core function of CST6 is to mediate pleiotropic effects across multiple systems by regulating protease-antiprotease homeostasis. Its functional specificity, encompassing tumor-suppressive *versus* oncogenic activities and homeostatic maintenance *versus* pathological progression, is governed by key “molecular switches,” including glycosylation, subcellular localization, upstream transcriptional regulation, and selective binding to target proteases. These regulatory elements form an integrated hierarchical network.

##### 2.3.4.1 Selective binding to target proteases and dynamic equilibrium

CST6 potently inhibits cathepsin L/V, moderately inhibits cathepsin B, and exhibits no inhibitory activity against cathepsin C via its N-terminal flexible loop and other functional domains. Meanwhile, CST6 blocks the oncogenic activity of legumain (LGMN), thereby regulating extracellular matrix remodeling and tumor invasion (Cheng et al., 2006; Zeeuwen et al., 2001; Zeeuwen et al., 2010; D’Costa et al., 2014; Wallin et al., 2017). The imbalance in the CST6/protease ratio contributes to pathogenesis. Upregulated CST6 combined with decreased LGMN causes endothelial dysfunction in preeclampsia (Botha et al., 2025), and loss of CST6 causes uncontrolled protease activity and promotes tumor progression in metastatic oral squamous cell carcinoma (Vigneswaran et al., 2006).

##### 2.3.4.2 Glycosylation modification

Glycosylation at Asn119 acts as a key switch that regulates protein secretion. This modification generates non-glycosylated (∼14 kDa) and glycosylated (∼17 kDa) isoforms without altering protease inhibitory activity (Lunde et al., 2017). The glycosylated isoform is preferentially secreted extracellularly and promotes proliferation in pancreatic ductal adenocarcinoma by inhibiting the pro-apoptotic function of cathepsin B (Hosokawa et al., 2008). In contrast, the non-glycosylated isoform is retained intracellularly and functions as a tumor suppressor in breast cancer (D’Costa et al., 2014).

##### 2.3.4.3 Subcellular localization

Intracellular CST6 exerts tumor-suppressive roles by modulating NF-κB pathway and cell cycle progression, such as EGR1, driving its intracellular expression in rhabdomyosarcoma (Mohamad et al., 2018) and inhibiting IKKβ/IκBα phosphorylation in cervical cancer cells (Petermann et al., 1982). Secreted CST6 contributes to remodeling of the tumor microenvironment. CST6 secreted by breast cancer cells suppresses osteoclast differentiation (Jin et al., 2012; Li et al., 2021).

##### 2.3.4.4 Upstream transcriptional regulatory network

CST6 function is predominantly governed by the core TBX2-EGR1-CST6 axis, in which TBX2 forms a co-repressor complex with EGR1 to suppress CST6 transcription, thereby activating LGMN and driving breast cancer cell proliferation (D’Costa et al., 2014; Paulus et al., 1995). In the absence of TBX2, EGR1 acts as a transcriptional activator of CST6, forming a tumor-suppressive axis in rhabdomyosarcoma (Mohamad et al., 2018). Additionally, single-nucleotide polymorphisms (SNPs) in the 5′UTR region of CST6 modulate antifungal drug susceptibility (Guo et al., 2020).

In summary, CST6 regulates protease homeostasis via a hierarchical cascade that includes target protease binding, glycosylation modulation, subcellular sorting, and upstream transcriptional regulation. This integrated regulatory mode serves as the molecular basis for its dual functional roles across multiple systems and provides a unified framework for resolving the functional paradox of CST6.

## 3 The role of CST6 in tumor-related diseases

### 3.1 The inhibitory effect of CST6 on tumor-related diseases

#### 3.1.1 Nasopharyngeal carcinoma

The incidence of nasopharyngeal carcinoma (NPC) is rare globally but highly prevalent in Southern China (Li et al., 2025a), with a high risk of infiltration and metastasis (Li et al., 2025b). Studies show that lncRNA EP300-AS1 regulates CST6 expression by targeting the transcription factor (TF)AP2C, affecting its binding to the CST6 promoter region, thereby inhibiting NPC cell proliferation, migration, and invasion while promoting apoptosis and G1 phase cell cycle arrest (Xing et al., 2025).

#### 3.1.2 Skin cutaneous melanoma

Melanoma, a highly aggressive skin cancer, accounts for approximately 20% of skin cancer cases (Eigentler et al., 2025; Flemban et al., 2025). Mass spectrometry-based proteomic analysis has revealed significant downregulation of CST6 during regional lymph node metastasis. Loss of CST6 expression may promote extracellular matrix degradation and lymph node invasion by dysregulating cysteine protease inhibition (Azimi et al., 2023). CST6 expression positively correlates with epithelial infiltration involved in epithelial-mesenchymal transition and proliferation. Combined evaluation of epithelial infiltration and CST6 expression may predict skin cutaneous melanoma (SKCM) prognosis (Xu et al., 2021). Cells exhibit low uptake of exogenous CST6; however, the high affinity of CST6 for LGMN allows minimal internalization to significantly inhibit LGMN activity and suppress malignant phenotypes in melanoma (Wallin et al., 2017). CST6 expression decreases significantly with increasing tumor thickness, triggering metastatic phenotypic switching. CST6 shows mutually exclusive expression with pro metastatic genes such as MMP14, suggesting that these opposing factors participate in a core regulatory network that promotes metastatic transition (Riker et al., 2008).

#### 3.1.3 Breast cancer metastasis

Breast cancer is the most common malignancy and the second leading cause of cancer-related deaths among women globally (Aosmali et al., 2025), accounting for 24.2% of global cancer cases (Li et al., 2025c). CST6 is endocytosed by osteoclasts to inhibit CTSB cysteine protease activity, promoting SPHK1 accumulation and blocking the RANKL-p38 pathway, thereby suppressing osteoclast differentiation in breast cancer bone metastasis (Li et al., 2021). CST6 is a key component of the long-isoform adenosine kinase (ADK-L)-driven oncogenic pathway; its low expression releases metastatic potential (Shamloo et al., 2019). Genome-wide expression profiling reveals that CST6 cooperates with CST1/2/4 to synergistically inhibit metastasis (Johnstone et al., 2018). Cystatin M is absent in metastatic cancer cells and is potentially linked to the progression of primary tumors to metastatic phenotypes (Sotiropoulou et al., 1997). CST6 methylation silencing leads to the loss of tumor-suppressive function, inability to inhibit target enzymes such as cathepsins, and promotion of tumor invasion and metastasis (Benezeder et al., 2017). Methylation rates are significantly elevated in metastatic breast cancer. CST6 markedly inhibits breast cancer cell proliferation, migration, and invasion, and suppresses osteoclast activation and reduces osteolytic bone metastasis (Jin et al., 2012).

#### 3.1.4 Cutaneous squamous cell carcinoma

Cutaneous squamous cell carcinoma exhibits comparatively more aggressive behavior in specific populations (Daniels et al., 2025). Proteomic analysis has revealed significant downregulation of CST6 in metastatic lesions, potentially due to loss of protease inhibitory function, leading to enhanced cell migration and suppressed apoptosis (Azimi et al., 2020).

#### 3.1.5 Rhabdomyosarcoma

Rhabdomyosarcoma is the most common soft tissue sarcoma in young individuals (Gupta et al., 2024). CST6 functions as a tumor suppressor under EGR1 transcriptional control, and its expression is modulated by the EGR1-TBX2 axis, exerting antitumor effects by inhibiting the cell cycle and enhancing apoptosis (Mohamad et al., 2018).

#### 3.1.6 Lung adenocarcinoma

Lung adenocarcinoma is the most common histological type of lung malignancy (Fu et al., 2024). Studies have shown that CST6 methylation rates are significantly higher in smokers than non-smokers, potentially promoting tumor progression by releasing the inhibition of cathepsins (Tessema et al., 2014).

#### 3.1.7 Renal cancer

The most common kidney cancer in adults is renal cell carcinoma (RCC), with clear cell histology accounting for approximately 75% of cases (Bergmann et al., 2025). Retrospective analysis indicates that CST6 itself does not directly suppress tumor function in renal cancer but serves as a candidate epigenetic biomarker for prognosis prediction (Peters et al., 2014). Exogenous restoration of CST6 expression significantly inhibits RCC cell proliferation (Morr et al., 2010).

#### 3.1.8 Prostate cancer

Prostate cancer is the second most common cancer in men (Almeeri et al., 2024). Normal prostate epithelium highly expresses CST6, while its downregulation in prostate cancer leads to the loss of inhibition against cancer cell proliferation and invasion (Pulukuri et al., 2009). Studies suggest that 25(OH)D_3_ may directly bind VDR without CYP27B1 hydroxylation to activate tumor-suppressive pathways, and that CST6 is potentially involved in the anti-proliferative effects of 25(OH)D_3_ (Munetsuna et al., 2014).

#### 3.1.9 Glioma

Glioma is the most common malignant central nervous system tumor in adults (Zhang et al., 2025a). This tumor is characterized by high invasiveness driven by complex and poorly understood tic and epigenetic alterations. CST6 is inactivated by DNMT/HDAC dual silencing in glioma, and functional restoration inhibits tumor growth (Kim et al., 2006). In normal brain tissue, CST6 is highly expressed in oligodendrocytes and moderately expressed in astrocytes. Promoter hypermethylation leads to CST6 loss in most gliomas, reducing inhibition of Cathepsin B and enhancing tumor invasiveness (Qiu et al., 2008).

#### 3.1.10 Cervical cancer

Primarily caused by persistent human papilloma virus infection, cervical cancer is a global public health issue (Mwangi et al., 2025). CST6 blocks IKKβ/IκBα phosphorylation, inhibits NF-κB nuclear translocation and pro-survival transcriptional activity, induces apoptosis, and synergistically suppresses Cathepsin L to inhibit its pro-invasive function. These mechanisms collectively form a tumor-suppressive network targeting the NF-κB pathway and protease activity (Petermann et al., 1982). The inhibitory role of CST6 in tumor-related diseases is summarized in Table 1.

**TABLE 1:** The inhibitory role of CST6 in tumor-related diseases.

No.	Disease	Mechanism of action	Study type	References
1	Nasopharyngeal carcinoma (NPC)	Inhibition of EP300-AS1 leads to decreased CST6 expression, which inhibits NPC cell proliferation, migration, and invasion, while promoting apoptosis, arresting the cell cycle in the G1 phase, and regulating the EMT process. CST6 is a key effector molecule downstream in this pathway.	*In vitro*, *in vivo*	Xing et al. (2025)
2	Melanoma (SKCM)	Proteomic analysis found CST6 is significantly downregulated in the regional lymph node metastasis stage; its loss may promote ECM degradation and lymph node invasion by relieving inhibition of cysteine proteases. CST6 expression positively correlates with epithelial infiltration involved in EMT and proliferation. Exogenous CST6 has low cellular uptake but high affinity for legumain; minimal internalization significantly inhibits legumain activity, blocking the malignant phenotype. Genomic analysis reveals CST6 expression decreases significantly with increasing tumor thickness, triggering a metastatic phenotypic switch; it is mutually exclusive with pro-metastatic genes like MMP14, forming a core regulatory network for metastasis.	*In vitro*, Clinical sample	Azimi et al. (2023), Xu et al. (2021), Wallin et al. (2017), Riker et al. (2008)
3	Breast cancer metastasis	CST6 is endocytosed into osteoclasts to inhibit CTSB cysteine protease activity, leading to SPHK1 accumulation and blockade of the RANKL-p38 pathway, thereby inhibiting osteoclast differentiation in bone metastasis. CST6 is a key component of the ADK-L-driven oncogenic pathway; its low expression releases metastatic potential. Genome-wide expression profiling shows CST6 cooperates with CST1/2/4 to suppress metastasis. Cystatin M is expressed in normal and primary cancer cells but lost in metastatic cells; this loss may be associated with progression to a metastatic phenotype. Methylation silencing of CST6 leads to loss of tumor suppressor function, inability to inhibit target enzymes like cathepsins, and promotion of invasion/metastasis; methylation rates are significantly higher in metastatic breast cancer. CST6 significantly inhibits the proliferation, migration, and invasion of breast cancer cells, inhibits osteoclast activation, and reduces osteolytic bone metastasis.	*In vitro*, *In vivo*, Clinical sample	Jin et al. (2012), Li et al. (2021), Shamloo et al. (2019), Johnstone et al. (2018), Sotiropoulou et al. (1997), Benezeder et al. (2017)
4	Cutaneous squamous cell carcinoma (cSCC)	Proteomic analysis found CST6 is significantly downregulated in metastases, potentially by losing its protease inhibitory function, leading to enhanced cell migration and inhibited apoptosis.	Clinical sample	Azimi et al. (2020)
5	Rhabdomyosarcoma (RMS)	CST6 acts as a downstream tumor suppressor gene transcriptionally regulated by EGR1; its expression is modulated by the EGR1-TBX2 interaction axis, exerting its tumor-suppressive effects by inhibiting the cell cycle and enhancing apoptosis.	*In vitro*	Mohamad et al. (2018)
6	Lung adenocarcinoma	CST6 methylation rates are significantly higher in lung adenocarcinomas from smokers than non-smokers, potentially promoting tumor progression by relieving inhibition of cathepsins.	*In vitro*, Clinical sample	Tessema et al. (2014)
7	Renal cancer	In non-clear cell renal cell carcinoma (nccRCC), CST6 itself was not shown to have a direct tumor suppressor function but serves as a candidate epigenetic biomarker for prognosis prediction. In renal cell carcinoma (RCC), exogenous restoration of CST6 expression significantly inhibits RCC cell proliferation.	*In vitro*, Clinical sample	Peters et al. (2014), Morr et al. (2010)
8	Prostate cancer	CST6 is highly expressed in normal prostate epithelium, inhibiting lysosomal cysteine protease activity, but is downregulated in prostate cancer, losing its inhibitory effect on cancer cell proliferation and invasion. Analysis suggests CST6 may be involved in the anti-proliferative effects of 25(OH)D_3_, which directly binds VDR to activate tumor suppressor pathways independently of CYP27B1 hydroxylation.	*In vitro*, *In vivo*, Clinical sample	Pulukuri et al. (2009), Munetsuna et al. (2014)
9	Glioma	CST6 is inactivated in glioma via dual DNMT/HDAC-mediated silencing; its functional restoration can inhibit tumor growth. CST6 expression is lost in most gliomas due to promoter hypermethylation, leading to loss of inhibition of Cathepsin B and enhanced tumor invasiveness.	*In vitro*, Clinical sample	Kim et al. (2006), Qiu et al. (2008)
10	Cervical cancer (CC)	CST6 blocks IKKβ/IκBα phosphorylation, inhibits NF-κB nuclear translocation and its pro-survival transcriptional activity; induces cell-autonomous and non-autonomous apoptosis, and synergistically inhibits cathepsin L, blocking its pro-invasive function. These actions form a tumor-suppressive network targeting the NF-κB pathway and protease activity.	Clinical sample	Petermann et al. (1982)

### 3.2 The role of CST6 in promoting tumor-related diseases

#### 3.2.1 High-grade serous ovarian carcinoma

High-grade serous ovarian carcinoma, the most common and aggressive type of epithelial ovarian cancer, is characterized by high recurrence rates (Tadić et al., 2024; Sensi et al., 2025). The S100A9^+^ tumor cell subtype highly expresses CST6 and nine other s, significantly correlating with poor patient prognosis. *In vivo* and *in vitro* knockout experiments confirmed CST6 as a key oncogenic factor in this subtype; CST6 silencing inhibits tumor growth and invasion (Xu et al., 2024).

#### 3.2.2 Multiple myeloma

MM is an incurable hematologic malignancy of plasma cells (Cai et al., 2025; Yan et al., 2025). IL5RA is significantly upregulated in myeloma and exhibits co-expression with the secretory protein CST6 (Xu et al., 2023). Notably, CST6 is elevated in a subset of MM patients without osteolytic lesions (OLs). This elevation inhibits MM bone disease by blocking osteoclast differentiation and function (Sun et al., 2024). High CST6 expression is significantly associated with the absence of bone lesions (i.e., OLs). CST6 exerts bone-protective effects in MM by dual inhibition of osteoclast differentiation and function via suppressing osteoclast-specific cathepsin K activity and blocking the RANKL downstream NF-κB/TRAF3 signaling pathway (Gai et al., 2022). CST6 may promote myeloma proliferation while inhibiting MM-induced OLs. Previous studies have also suggested that CST6 is a potential common regulator of sarcopenia and osteoporosis and may influence the progression of both diseases (Liu et al., 2023).

#### 3.2.3 Triple-negative breast cancer

Triple-negative breast cancer (TNBC) is an aggressive breast cancer subtype with a high recurrence risk and poor clinical outcomes (Malvi et al., 2025; Wen et al., 2025). CST6 acts as a downstream effector of nuclear ADK-L. The expression of CST6 is positively regulated by ADK-L. ADK-L is specifically highly expressed in cancer tissues, and its knockdown inhibits cancer cell proliferation and migration (Shamloo et al., 2019). CST6 expression is significantly higher in TNBC tissues than that in adjacent normal tissues and is strongly associated with a high risk of lymph node metastasis. High CST6 expression is an independent poor prognostic factor for disease-free survival in TNBC (Li et al., 2018).

#### 3.2.4 Hepatocellular carcinoma

Hepatocellular carcinoma (HCC) is a highly heterogeneous malignant tumor with persistently poor long-term survival outcomes (Zheng et al., 2025a; Liu et al., 2025). Unlike non-tumor hepatitis or cirrhosis in hepatitis B virus/hepatitis C virus (HBV/HCV)-infected backgrounds, CST6 is incorporated into an upstream regulatory network activated by AMELY, promoting malignant cell transformation (Qi et al., 2013). As a core downstream effector of the PTHLH signaling pathway, CST6 induces apoptosis by inhibiting peptidase activity and synergistically suppressing Wnt signaling and fatty acid synthesis to drive malignant progression (Huang et al., 2012). Its expression level strongly correlates with recurrence-free survival and overall survival in patients with HBV-HCC (Zhou et al., 2019). CST6 may serve as a prognostic biomarker for HCC. High expression of CST6 has been associated with poor patient outcomes—consistent with previous studies identifying CST6 as a candidate molecular target for pancreatic cancer therapy (Hosokawa et al., 2008).

#### 3.2.5 Pancreatic ductal adenocarcinoma

Pancreatic ductal adenocarcinoma (PDAC) is a rare and aggressive cancer which is often diagnosed late in the disease and has low survival rates (Zheng et al., 2025b; Jiang et al., 2025). CST6 drives tumor proliferation by blocking the pro-apoptotic function of Cathepsin B via its glycosylated protease inhibition domain. CST6 is specifically highly expressed in PDAC but not present in normal pancreatic tissues. The knockdown of siRNA significantly inhibits growth (Hosokawa et al., 2008).

#### 3.2.6 Papillary thyroid carcinoma

The global incidence of thyroid cancer has risen sharply (Chen and Haymart, 2025). Papillary thyroid carcinoma (PTC) is the most common type of thyroid carcinoma. PTC is characterized by early lymph node metastasis and has distinct biological behavior, clinical management, and prognosis which differentiate it from other subtypes (Zhang et al., 2025b). CST6 is specifically highly expressed in PTC and positively correlates with lymph node metastasis (Oler et al., 2008).

#### 3.2.7 Oropharyngeal squamous cell carcinoma

Oropharyngeal squamous cell carcinoma (OSCC) is an invasive epithelial tumor with high metastasis and recurrence rates and limited treatment efficacy (Huo et al., 2025; Wang et al., 2025). CST6 is abnormally highly expressed in OSCC metastases, with expression levels 40-fold higher in metastatic cell lines than those in primary tumor cell lines. It promotes tumor cell survival during metastasis by inhibiting TNF-α-induced apoptosis via suppression of Cathepsin B enzyme activity (Vigneswaran et al., 2003). Notably, in metastatic oral cancer cells, CST6 directly blocks cell invasion and motility by inhibiting Cathepsin B/L and LGMN protease activity. The loss of CST6 leads to uncontrolled protease activity, driving hyperproliferation and metastatic potential (Vigneswaran et al., 2006). These studies suggest that high CST6 expression may enhance primary tumor growth while inhibiting metastatic spread (Huo et al., 2025; Wang et al., 2025; Vigneswaran et al., 2003; Vigneswaran et al., 2006).

#### 3.2.8 Dual role of CST6 in gastric cancer

Gastric cancer (GC) is a highly prevalent and lethal malignancy worldwide (Ma et al., 2025a). Gastric adenotumor is a rare subtype of gastric tumor (Qin et al., 2025). CST6 is significantly overexpressed in patients with high-risk stomach adenocarcinoma (STAD), potentially promoting tumor progression by regulating metabolic-immune microenvironment crosstalk, demonstrating strong predictive potential for STAD outcomes (Li et al., 2023). Necroptosis plays a crucial role in GC development, prognosis, and in the shaping of the tumor immune microenvironment. CST6 acts as an oncogenic factor in a necroptosis-related (NRG) prognostic model (NRGPI). In high-risk NRGPI groups, CST6 is synergistically upregulated with TGF-β/WNT pathways, promoting Treg cell and M2 macrophage infiltration to form an immune “cold tumor” microenvironment. High CST6 expression in GC cell lines enhances immunosuppression, activates para-inflammatory and type II interferon pathways, and exacerbates tumor-related inflammatory damage (Khan et al., 2022). CST6 serves as a core oncogenic factor in a CpG island methylator phenotype-related six-prognostic signature for GC, driving immunosuppressive microenvironments and pro-metastatic pathways to worsen patient survival (Zeng et al., 2020). Paradoxically, the discovery of CST6’s oncogenic or tumor-suppressive role in gastric malignancies contrasts with earlier findings. Previous studies indicated that CST6 silencing in GC due to promoter hypermethylation, resulted in a loss of CST6 expression in 70% of cases and a loss of methylation in 55%. Moreover, patients whose DNA had undergone methylation had significantly shorter survival. Demethylating agents can restore CST6 expression, thereby establishing it as a tumor suppressor in GC (Chen et al., 2010). The role of CST6 in promoting tumor-related diseases is summarized in Table 2.

**TABLE 2:** The role of CST6 in promoting tumor-related diseases.

No.	Disease	Mechanism of action	Study type	References
1	High-grade serous ovarian carcinoma (HGSC)	The S100A9, tumor cell subtype highly expresses CST6 and other genes, which is significantly associated with poor patient prognosis; *in vivo* and *in vitro* knockout experiments confirm that CST6 acts as a key pro-cancer factor in this subtype, and its silencing inhibits tumor growth and invasion.	*In vitro*, *In vivo*, Clinical sample	Xu et al. (2024)
2	Multiple myeloma (MM)	Tumor-promoting Effect:​ IL5RA is significantly upregulated in myeloma and shows a co-expression relationship with the secretory protein gene CST6. CST6 can promote myeloma proliferation.	*In vitro*, *In vivo*, Clinical sample	Xu et al. (2023), Sun et al. (2024), Gai et al. (2022)
		Bone-protective effect (inhibiting bone Disease):​​ CST6 is elevated in a subgroup of MM patients without osteolytic lesions (OL); it inhibits MM bone disease by blocking osteoclast differentiation and function. High CST6 expression is significantly associated with the absence of OLs. It dually inhibits osteoclast differentiation and function by inhibiting osteoclast-specific cathepsin K activity and blocking the RANKL downstream NF-κB/TRAF3 signaling pathway.	*In vitro*, *In vivo*, Clinical sample	
3	Triple-negative breast cancer (TNBC)	CST6 acts as a downstream effector of long-form adenosine kinase (ADK-L), and its expression is positively regulated by ADK-L. CST6 expression is significantly higher in TNBC tissues than in adjacent normal tissues and is significantly associated with a high risk of lymph node metastasis. High CST6 expression is an independent poor prognostic factor for disease-free survival in TNBC.	*In vitro*, Clinical sample	Shamloo et al. (2019), Li et al. (2018)
4	Hepatocellular carcinoma (HCC)	CST6 is included in an upstream regulatory network activated by AMELY, promoting malignant cell transformation. As a core downstream effector of the PTHLH signaling pathway, its high expression level is strongly correlated with shorter recurrence-free survival (RFS) and overall survival (OS) in HBV-HCC patients.	Clinical sample	Qi et al. (2013), H et al. (2012), Zhou et al. (2019)
5	Pancreatic ductal adenocarcinoma (PDAC)	CST6 drives tumor proliferation by using its glycosylated protease inhibition domain to block the pro-apoptotic function of Cathepsin B. CST6 is specifically highly expressed in PDAC but negative in normal pancreas; siRNA knockdown significantly inhibits growth.	*In vitro*, *In vivo*	Hosokawa et al. (2008)
6	Papillary thyroid carcinoma (PTC)	CST6 is specifically highly expressed in PTC and is positively correlated with lymph node metastasis.	Clinical sample	Oler et al. (2008)
7	Oropharyngeal squamous cell carcinoma (OSCC)	Tumor-promoting Effect:​ CST6 is abnormally highly expressed in OSCC metastases (40 times higher in metastatic cell lines than in primary tumor cell lines). It promotes survival of metastatic tumor cells by inhibiting Cathepsin B enzyme activity to block TNF-α-induced apoptosis.	*In vitro*, Clinical sample	Vigneswaran et al. (2003), Vigneswaran et al. (2006)
		Metastasis-inhibiting Effect:​ Interestingly, another study found that loss of CST6 expression in metastatic oral cancer cells leads to uncontrolled protease activity, driving hyperproliferation and surging metastatic potential, suggesting it directly blocks invasion and motility by inhibiting Cathepsin B/L and legumain protease activity.	*In vitro*	

## 4 The role of CST6 in non tumor diseases

### 4.1 Skin homeostasis

CST6 is a specific secretory protein in human skin and sweat glands, serves to maintain normal epidermal differentiation, and is secreted into sweat for immune defense (Zeeuwen et al., 2001). Loss of CST6 leads to excessive activation of Cathepsin L, disrupting stratum corneum morphology, causing epidermal barrier collapse, and resulting in abnormal corneal epithelial keratinization (Zeeuwen et al., 2010). The CST6-Cathepsin L-TGase3 pathway drives terminal differentiation of skin appendages (hair follicles and nail plates) by precisely inhibiting Cathepsin L and activating TGase3-mediated cross-linking of structural proteins (Cheng et al., 2009). These disorders arise from genetic alterations that disrupt normal epidermal differentiation. These disorders include non-syndromic and syndromic subtypes with broader skin involvement or palmoplantar keratoderma (Akiyama et al., 2025). In a reported case of hereditary keratoderma, a homozygous loss-of-function mutation in the CST6 was identified as the underlying cause. The mutated CST6 protein lost its ability to inhibit cathepsins, disrupting the skin protease-antiprotease balance. This led to disrupted keratinocyte differentiation and barrier dysfunction in mouse models, manifesting as dry skin, desquamation, and abnormal keratinization (Wang et al., 2023). KFSD is another rare tic skin disorder in which aberrant subcellular localization of CST6 protein impairs its inhibition of cathepsins (CTSL/CTSV) and causes dysregulated transglutaminase activation, resulting in defective terminal epidermal differentiation and impaired hair follicle development (Eckl et al., 2021). Autosomal recessive hypotrichosis is caused by a homozygous variant introducing a premature stop codon in exon 2 of CST6. Individuals with this disease may present with sparse hair, eczema, blepharitis, photophobia, and sweating disorders. Studies have shown that the recombinant mutant CST6 protein failed to inhibit its three target proteases in enzymatic assays, as the protease inhibitor binding sites for LGMN and Cathepsins L/V were disrupted in the p. Gln121* variant. CST6 plays a critical role in epidermal homeostasis and hair follicle morphosis (van den Bogaard et al., 2019). Ichthyosis, Alopecia, and Keratopathy: Studies in mouse and human skin models have revealed that CST6 maintains epidermal keratinization and hair follicle morphology by inhibiting cathepsins. Its loss leads to ichthyosis-like phenotypes and lethality in mice. In humans, CST6 is essential for epidermal stratification, and its deficiency may cause embryonic lethality (Oortveld et al., 2017; Zeeuwen et al., 2002a; Jansen et al., 2012).

Psoriasis is a common chronic papulosquamous skin disease currently considered incurable (Griffiths et al., 2021). CST6 has been identified as a key associated with impaired mitophagy in psoriasis. Downregulation of CST6 significantly correlates with skin barrier dysfunction and hyperactivation of pro-inflammatory and pro-proliferative signaling pathways in psoriasis. CST6 maintains barrier function in healthy skin, and its reduced expression in psoriasis may diminish these protective effects (Yu et al., 2025). The systemic inflammatory cytokine IL-17A directly downregulates CST6, interfering with normal keratinocyte differentiation, inhibiting granular layer formation, disrupting skin barrier function, and ultimately contributing to characteristic psoriatic lesions (Sato et al., 2020). During inflammation, CST6 extends to the spinous layer to inhibit excessive proteolysis, and its expression marks re-epithelialization maturity in wound healing (Zeeuwen et al., 2002b). Lyme disease (LD), caused by spirochete *Borrelia burgdorferi* infection, initially presents as a circular, inflamed skin lesion called erythema migrans (Zhou et al., 2020). LD can lead to arthritis, dermatological changes, and various neurological and cardiac manifestations (Sigal, 2025). Serum CST6 levels change significantly in the early stages of LD and are potentially involved in the cutaneous immune response (Zhou et al., 2020).

### 4.2 Reproduction and pregnancy

Preeclampsia is a multisystem syndrome associated with inflammation, oxidative and endoplasmic reticulum stress, and angiogenic dysfunction (Roberts et al., 2021). Preeclampsia is caused by placental insufficiency; the placenta significantly upregulates CST6 expression under hypoxic conditions. Both CST6 mRNA in the placenta and maternal blood CST6 protein levels are markedly elevated in pre-eclamptic pregnancies. These levels are detectable even before clinical symptoms appear, offering early predictive value. Elevated circulating CST6 may contribute to the core pathological processes of maternal vascular endothelial damage and multi-organ dysfunction in preeclampsia by regulating its target asparaginyl endopeptidase and exacerbating TNFα-induced endothelial cell dysfunction (Botha et al., 2025). In a bovine oviduct organoid model, heat stress (HS) significantly upregulated CST6 expression; Pathway analysis confirmed that the heat stress (HS)-activated genes including CST6, COX1, ACTB, TPT1 and HSPB1 collectively mediate HS-induced impairment of mammalian reproductive function by regulating cellular senescence and the p53/TGF-β signaling pathways (Menjivar et al., 2023). A study comparing uterine tissue transcriptomes from non-pregnant goats with those at 15 and 19 days of pregnancy revealed CST6 involvement in embryo implantation regulation (Zhao et al., 2023). Endometrial expression profiling in cattle at 15–17 days of pregnancy *versus* non-pregnant/cyclic groups showed significant upregulation of CST6 during pregnancy; this finding is potentially related to embryo implantation (Adhik et al., 2022).

### 4.3 Diabetes mellitus (DM)

DM is a complex metabolic disorder characterized by hyperglycemia and is primarily caused by insufficient insulin secretion or the development of insulin resistance (Ke et al., 2025). CST6 expression is elevated in plasma/serum-derived extracellular vesicles from patients with type 2 DM. CST6, may influence disease progression through the inflammatory response, complement activation, and platelet activation pathways (Khan et al., 2022). In diabetic retinopathy (DR), CST6 may function in tissue repair or stress response and is particularly associated with pan-retinal photocoagulation therapy. Proteomic analysis has identified CST6 as one of the significantly altered marker proteins following laser treatment for DR (Oh et al., 2025).

### 4.4 Chronic kidney disease

Chronic kidney disease (CKD) is a progressive condition associated with declining renal function (Oh et al., 2025; Bakinowska et al., 2024) and is linked to higher mortality rates (Ma et al., 2025b). CST6 protein shows early changes in expression during renal function decline. Its diagnostic performance (AUC = 0.81) significantly exceeds that of the traditional estimated glomerular filtration rate (eGFR) (Khoza et al., 2025). Initial urinary proteomic screening has suggested that CST6, combined with FABP1, FABP3, and B2M, may distinguish patients at high risk for acute kidney injury requiring hemodialysis. However, CST6 and B2M were not validated by Western blot in that study, excluding CST6 as a reliable marker for this purpose (Dihazi et al., 2016).

### 4.5 Idiopathic pulmonary fibrosis

Pulmonary fibrosis (PF) is a chronic inflammatory interstitial lung disease (Salem et al., 2025). Idiopathic pulmonary fibrosis (IPF) is a devastating form of interstitial lung disease with poor survival outcomes (Khalid et al., 2025). CST6 is one of seven core predictive marker s. Its expression level is significantly altered in patients with IPF. CST6 may participate in the fibrotic process by regulating extracellular matrix remodeling or protease inhibition (Luo et al., 2023).

### 4.6 Neuropsychiatric systemic lupus erythematosus

Neuropsychiatric systemic lupus erythematosus (NPSLE) is a severe complication of systemic lupus erythematosus (SLE) affecting the central or peripheral nervous system (Guan et al., 2025). Large-sample data identified CST6, together with KLK5 and TCN2, as a core triple marker for diagnosing NPSLE, effectively distinguishing NPSLE from rSLE. CST6 levels correlate significantly with NPSLE disease activity. Synchronous changes in CST6 levels in cerebrospinal fluid and hippocampal tissue have suggested CST6 as a reliable indicator of intracerebral pathological processes (Ni et al., 2023).

### 4.7 Cardiovascular disease

Cardiovascular diseases, caused by a combination of genetic and environmental risk factors (Yun et al., 2022), are a leading cause of global morbidity and mortality (Soppert et al., 2020). CST6, as a core plasma protein, together with the obesity-related factor leptin, the extracellular matrix protein proline arginine-rich end leucine-rich repeat protein, and the neuroendocrine protein chromogranin B, forms a key predictive protein panel consistent across models for predicting carotid intima-media thickness (cIMT) measurements (Chen et al., 2024a).

### 4.8 Bladder dysfunction

Bladder dysfunction is defined as a state of complete anuria or oliguria lasting at least 6 months (Wadie et al., 2024). This dysfunction refers to a loss of the bladder’s normal storage and voiding functions due to a lack of urine. In patients with end-stage renal disease and in rabbit models mimicking this process, downregulation of CST6 in the detrusor muscle has been closely associated with structural and functional deterioration of the bladder. This manifestation may be irreversible even after urine flow is restored. This suggests that CST6 is essential for maintaining the structural and functional integrity of the bladder. Loss of cystatin function may disrupt the proteolytic balance in the bladder wall, thereby affecting smooth muscle contraction and extracellular matrix homeostasis (Kisou et al., 2020). The role of CST6 in non tumor diseases is summarized in Table 3.

**TABLE 3:** The role of CST6 in non tumor diseases.

No.	Disease/Physiological Process	Mechanism of action	Study type	References
1	Skin homeostasis	Functions as a skin-specific secretory protein, maintaining normal epidermal differentiation, barrier function, and immune defense by inhibiting exogenous Cathepsin B and participating in transglutaminase-mediated keratin cross-linking.	*In vitro*, Clinical sample	Zeeuwen et al. (2001)
2	Epidermal differentiation disorders (EDD) and related genetic skin diseases	Loss-of-function mutations (e.g., c.251G>A) cause CST6 to completely lose its ability to inhibit cathepsins, disrupting the protease-antiprotease balance. This leads to disordered keratinocyte differentiation, barrier function collapse, abnormal skin keratinization, and hair follicle development disorders.	*In vitro*, *In vivo*, Clinical sample	Zeeuwen et al. (2010), Cheng et al. (2009), Akiyama et al. (2025), Wang et al. (2023), Eckl et al. (2021), van den Bogaard et al. (2019), Oortveld et al. (2017), Zeeuwen et al. (2002a), Jansen et al. (2012)
3	Psoriasis	Downregulation of expression is associated with skin barrier dysfunction and overactivation of pro-inflammatory/proliferative pathways. The inflammatory cytokine IL-17A can directly downregulate CST6, interfering with keratinocyte differentiation and damaging barrier function. Its expression serves as a marker for mature re-epithelialization during wound healing.	*In vitro*, *In vivo*, Clinical sample	Yu et al. (2025), Sato et al. (2020), Zeeuwen et al. (2002b)
4	Lyme disease	Serum levels change significantly in the early stages of the disease, potentially involved in the skin immune response.	Clinical sample	Zhou et al. (2020)
5	Reproduction and pregnancy (e.g., preeclampsia)	Placental expression is significantly upregulated under hypoxia. CST6 mRNA in the placenta and protein levels in maternal blood are significantly elevated, offering early predictive value. Elevated circulating CST6 may contribute to the pathology by regulating its target asparagine endopeptidase and exacerbating TNF伪-induced endothelial cell dysfunction. Also involved in heat stress-related damage to reproductive function and the regulation of embryo implantation.	*In vitro*, Clinical sample	Botha et al. (2025), Menjivar et al. (2023), Zhao et al. (2023), Adhik et al. (2022)
6	Diabetes mellitus	Expression is elevated in plasma/serum extracellular vesicles of type 2 diabetes patients, potentially involved in disease progression via inflammation and complement activation pathways. In diabetic retinopathy, it is a significantly altered marker protein after laser treatment, possibly involved in tissue repair or stress response.	Clinical sample	Khan et al. (2022), Oh et al. (2025)
7	Chronic kidney disease (CKD)	Shows differential expression in early renal function decline, demonstrating good performance in distinguishing early renal impairment. However, it was not validated as a reliable biomarker for acute kidney injury requiring dialysis in one study.	Clinical sample	Khoza et al. (2025), Dihazi et al. (2016)
8	Idiopathic pulmonary fibrosis (IPF)	As one of the core predictive markers, its expression level changes significantly in IPF patients, potentially involved in the fibrotic process by regulating ECM remodeling or protease inhibition.	Clinical sample	Luo et al. (2023)
9	Neuropsychiatric systemic lupus erythematosus (NPSLE)	Forms a core triple biomarker panel with KLK5 and TCN2 for diagnosing NPSLE, effectively distinguishing it from non-NPSLE patients. Its levels correlate with disease activity, indicating it is a reliable indicator of intracerebral pathology.	Clinical sample	Ni et al. (2023)
10	Cardiovascular disease	As a core plasma protein, it forms a key predictive panel with LEP, PRELP, etc., for predicting carotid intima-media thickness (cIMT).	Clinical sample	Chen et al. (2024a)
11	Bladder dysfunction	Expression is downregulated in dysfunctional bladders and is closely associated with structural and functional degeneration. Loss of cystatin function may disrupt proteolytic balance in the bladder wall, affecting smooth muscle contraction and ECM homeostasis.	*In vivo*, Clinical sample	Kisou et al. (2020)

## 5 Research on CST6 as a target for clinical diagnosis and treatment

### 5.1 Diagnostic biomarkers

#### 5.1.1 Liquid biopsy

In oncological liquid biopsies, detection of CST6 promoter methylation in circulating tumor cells and cell-free DNA (cfDNA) holds significant value. In breast cancer, the methylation status of CST6 is an independent prognostic factor for low circulating tumor cell survival and shorter overall survival (Chimonido et al., 2017). Thus, CST6 is an effective marker for monitoring recurrence in plasma cfDNA (Chimonidou et al., 2013). Other studies have found CST6 in multi-methylation liquid biopsy models including SOX17and BRMS1 (Parisi et al., 2016; Li et al., 2015). In non-oncological liquid biopsies, serum CST6 levels serve as an early diagnostic marker for LD (Zhou et al., 2020); a panel of three hub proteins (CST6, TCN2, KLK5) effectively distinguishes NPSLE from SLE, with CST6 acting as a cerebrospinal fluid biomarker highly correlated with disease activity (Ni et al., 2023). In addition, maternal blood CST6 levels are a potential marker for preeclampsia (Botha et al., 2025).

#### 5.1.2 Tissue/body fluid protein expression

In high-grade serous ovarian cancer, CST6 is a key marker of the S100A9^+^ pro-metastatic subtype, promoting abdominal/pelvic metastasis by driving tumor invasion and remodeling the immune microenvironment (Xu et al., 2024). Low or absent CST6 expression is a poor prognostic indicator in GC and TNBC (Zeng et al., 2020; Li et al., 2018; Shamloo et al., 2019), while hypermethylation in renal cancer correlates with poor prognosis and resistance to VEGF-targeted therapy (Peters et al., 2014), enabling prognostic stratification. In cervical cancer, CST6 mediates fatty acid metabolism-immune microenvironment crosstalk, inhibits immune escape, and enhances chemosensitivity. It is a core factor in prognostic models, offering risk stratification and novel strategies for combination therapy targeting metabolic-immune pathways (Zeng et al., 2022). CST6 has been identified as a significant marker for Stage IV colorectal cancer, with expression levels changing markedly during progression (Palaniappan et al., 2024). Recurrent colorectal cancers show significantly higher CST6 protein expression scores than those in non-recurrent cases. As an independent risk factor, high CST6 expression correlates significantly with poor tumor differentiation and directly promotes postoperative recurrence in early colon cancer (Zhu et al., 2024). In gastric, colon, and pancreatic cancers, CST6 expression and its associated signatures are effective tools for subtyping and predicting immunotherapy response or recurrence risk (Khan et al., 2022; Zhu et al., 2024; Chen et al., 2024b). Timely management of CKD is crucial to prevent end-stage renal disease, and accurate prediction of CKD progression is vital for early intervention and personalized therapy (Toan Duong et al., 2025). In CKD, urinary CST6 protein serves as an eGFR staging marker, providing a highly sensitive, non-invasive screening tool that overcomes the insensitivity of current eGFR and proteinuria tests for early CKD (Khoza et al., 2025). Plasma proteins such as CST6 can act as novel non-invasive cardiovascular risk assessment markers for long-term prediction of cIMT, addressing the shortcomings of traditional risk factors in long-term cIMT prediction (Chen et al., 2024a).

### 5.2 Novel therapeutic strategies

#### 5.2.1 Restoring expression and function

Demethylation Therapy: Studies show that CST6 overexpression inhibits tumor cell migration, invasion, and proliferation *in vitro* and in animal models (Hosokawa et al., 2008; Song et al., 2006), representing a potential therapy strategy. Using DNA methyltransferase inhibitors (e.g., 5-aza-CdR) can reverse CST6 methylation silencing, induce its re-expression in glioma and breast cancer models, and exert tumor-suppressive effects via demethylation (D’Costa et al., 2014; Qiu et al., 2008; Rivenbark et al., 2006).

Targeting Regulatory Pathways: Targeting the inhibition of the oncogenic factor TBX2 or activating its upstream regulator EGR1 can relieve its suppression on CST6, offering a strategy for treating tumors with high TBX2 expression (D’Costa et al., 2014; Mohamad et al., 2018). The high efficacy of biologics targeting IL-17A (e.g., Secukinumab, Ixekizumab) in psoriasis treatment is attributed to both inflammation suppression and direct promotion of epidermal structure and function recovery (Sato et al., 2020).

#### 5.2.2 Exogenous supplementation

Bone metastasis is a common complication of breast cancer and is associated with disease progression and mortality (Jin et al., 2012). Most patients with MM develop OLs. Bisphosphonates such as zoledronic acid are used to improve bone resorption; however, novel anti-resorptive agents are needed owing to the risks of severe side effects and lack of repair in existing lesions (Gai et al., 2024). Recombinant CST6 protein significantly increases estrogen receptor alpha (ERα) expression and intracellular estrogen concentration in osteoclast precursors, inhibiting their maturation (Gai et al., 2024). In models of breast cancer bone metastasis and MM bone disease, recombinant CST6 or its active peptides effectively inhibited osteoclast differentiation, reduced bone destruction, and showed potential for treating OL (Gai et al., 2024; Jin et al., 2012; Li et al., 2021; Gai et al., 2022). Ketamine inhibits the transcriptional repression of CST6 by SRC via EGR1 release. Overexpression of EGR1 or CST6 can block the oncogenic effects of SRC and restore ketamine’s inhibition of cancer cells and osteoclast activity (Zhang et al., 2024).

#### 5.2.3 Overcoming drug resistance

C. glabrata is an opportunistic human fungal pathogen with a recent increasing incidence of infection. An SNP variant in the 5′UTR region of the CST6 is significantly associated with fluconazole resistance in C. glabrata. This variant may affect the ERG sterol pathway by regulating the CST6 expression and synergizing with subtelomeric copy number variation to drive pathogen adaptive evolution. This finding provides a molecular tool for rapid resistance detection and precise medication in candidiasis (Guo et al., 2020). *In vitro* microevolution experiments found that when the transcription factor UPC2A is inactivated, its mutant (upc2AΔ) becomes highly sensitive to fluconazole; concurrent deletion of the transcription factor CST6 reverses this hypersensitivity and restores the resistant phenotype (Ollinger et al., 2025). Fungal CST6 (non-human homolog) acts as a negative transcriptional regulator of the ergosterol biosynthesis pathway, reducing fungal sensitivity to fluconazole by inhibiting ERG expression. Small molecule inhibitors targeting CST6 can block its negative regulatory function, restore ERG expression and toxic sterol accumulation, thereby overcoming intrinsic resistance (Ollinger et al., 2021). The CST6 homolog in C. glabrata has been identified as a key regulator of fluconazole susceptibility and resistance (Guo et al., 2020).

## 6 Controversies and future directions

### 6.1 Core contradictions and focus

The dual roles of CST6, including both cancer promotion and suppression, represent a central controversy and challenge in current research primarily attributed to the following variables. 1) Tissue Specificity and Microenvironment Heteroity: The function of CST6 highly depends on the cell type which is expressing it, the state of the tumor microenvironment, and interacting molecules. For example, elevated CST6 in hypoxic placental environments reflects pathological conditions (Botha et al., 2025). Moreover, its glycosylation status may influence pro-proliferative functions in specific pancreatic cancer cell lines (Hosokawa et al., 2008). In addition, its high expression in TNBC is associated with poor prognosis and may involve complex interactions with other molecules or specific signaling contexts (Shamloo et al., 2019; Li et al., 2018). 2) Complexity of Regulatory Networks: CST6 acts as a node intersecting multiple signaling pathways. Its position upstream or downstream in these pathways, along with synergistic or antagonistic interactions with other molecules, leads to divergent biological outcomes. 3) Loss of Function Versus Gain of Function: In most tumors, epigenetic silencing and loss of CST6 function drive cancer promotion. Several studies reporting the “pro-tumor” effects of CST6 suggest that CST6 is a bystander marker of specific pathological states (e.g., preeclampsia, S100A9^+^ ovarian cancer subtype) rather than a direct driver. Other studies have suggested a context-dependent gain-of-function effect. 4) Discrepancies Between Experimental Models and Clinical Samples: Findings from in vitrocell-line studies may not have fully captured the complexity of the *in vivo* tumor microenvironment and patient heteroity.

### 6.2 Emerging research fields

#### 6.2.1 Functional heteroity of CST6

Utilizing single-cell sequencing, spatial transcriptomics, and other advanced technologies to deeply investigate the expression patterns, regulatory networks, and specific functions of CST6 across different cell types and regions of the tumor microenvironment can serve to clarify the cellular and molecular basis of its contradictory roles.

#### 6.2.2 Mechanisms of immune microenvironment regulation

This field of research involves the exploration of the mechanisms of CST6 in the modulation of the tumor immune microenvironment—such as T cell infiltration, macrophage polarization, and immune checkpoint expression—through effects on protease activity, cytokine release, and antigen presentation, as well as its role in immune responses in autoimmune diseases. The function of CST6 in the immune typing of cervical and gastric cancers suggests its role as a key node in immune regulation (Khan et al., 2022; Zeng et al., 2022).

#### 6.2.3 Innovative therapies targeting CST6

CST6 Agonists and Stabilizers: For tumors with a loss of CST6 expression or function (e.g., bone metastases, methylation-silenced tumors), the future development of small-molecule agonists, stabilizers, or protein delivery systems to overcome the limitations of recombinant protein applications could offer promising treatment options. Combination Therapies: These therapies integrate strategies to restore CST6 function with immune checkpoint inhibitors, targeted therapy, radiotherapy, or chemotherapy. Further research is required to elucidate the synergistic effects. Novel Drugs Based on Resistance Mechanisms: Specific drugs are being developed to target CST6-related resistance pathways in *Candida* species.

#### 6.2.4 Translational research on CST6 in non-oncological diseases

Such research incorporates advanced preclinical and clinical translation of recombinant CST6 for treating osteolytic bone diseases. The mechanisms of CST6 in metabolic and neuropsychiatric disorders must be investigated further to develop diagnostic and interventional strategies. Moreover, research is needed to fully explore the reparative potential of CST6 in conditions such as bladder dysfunction and hair loss syndromes.

#### 6.2.5 Optimization of CST6 as a cross-disease biomarker

This field of research serves to integrate multi-omics data (genomics, epigenomics, proteomics) to construct and validate multimodal diagnostic, prognostic, and predictive models based on CST6 and its regulatory networks, enhancing its precision and clinical utility in liquid biopsies, tissue typing, and treatment response monitoring.

## 7 Conclusion

CST6 is a secreted cysteine protease inhibitor with a core structural domain composed of β-sheets and an α-helix. CST6 features key sites that bind to the active centers of target enzymes. It exhibits a dual role in tumors. For example, in breast cancer metastases and NPC, CST6 acts as a tumor suppressor by inhibiting NF-κB nuclear translocation and modulating the TBX2-LGMN apoptosis axis. In S100A9^+^ subtype ovarian cancer and TNBC, the high expression of CST6 drives proliferation, invasion, and immune microenvironment dysregulation, demonstrating its pro-tumor effects. This functional paradox stems from differences in tissue microenvironments and regulatory networks. In non-oncological diseases, the dysregulation of CST6 can drive multisystem pathologies such as psoriasis, diabetes, and preeclampsia. Urinary CST6 serves as an early sensitive biomarker for CKD. Cerebrospinal fluid CST6 levels correlate with disease activity in NPSLE. CST6 also contributes to pulmonary fibrosis and cardiovascular risk prediction. Diagnostically, CST6 levels in serum, urine, and tissues function as diagnostic or prognostic markers for various tumors and non-neoplastic diseases. Therapeutic strategies involving CST6 include epigenetic drugs to restore its expression, upstream-targeting agents, and exogenous recombinant proteins to inhibit bone destruction. The development of epigenic drugs and exogenous recombinant proteins in research currently remains at the animal model or preclinical stage. Future research should focus on elucidating the mechanisms underlying the functional contradictions of CST6 and exploring its role in immune-protease crosstalk in autoimmune diseases, developing agonist-based combination therapies, and expanding its utility in areas such as bone metabolic disorders. The ultimate goal is to construct a precision medicine model based on the CST6 regulatory network.

## Nomenclature

CST6: Cystatin 6
MM: Multiple myeloma
OVX: Ovariectomy
TNF: Tumor necrosis factor
rmCst6: Recombinant mouse CST6
ERα: Estrogen receptor alpha
CTSB: Cathepsin B
LGMN: Legumain
KFSD: Keratosis follicularis spinulosa decalvans
NPS: Nasopharyngeal carcinoma
TF: Transcription factor
SKCM: Skin cutaneous melanoma
RCC: Renal cell carcinoma
OLs: Osteolytic lesions
TNBC: Triple-negative breast cancer
ADK-L: Long-isoform adenosine kinase
HCC: Hepatocellular carcinoma
HBV: Hepatitis B virus
HCV: Hepatitis C virus
PDAC: Pancreatic ductal adenocarcinoma
PTC: Papillary thyroid carcinoma
OSCC: Oropharyngeal squamous cell carcinoma
GC: Gastric cancer
STAD: Stomach adenocarcinoma
NRG(PI): Necropsis-related prognostic model
LD: Lyme disease
HS: Heat stress
DM: Diabetes mellitus
DR: Diabetic retinopathy
CKD: Chronic kidney disease
eGFR: Estimated glomerular filtration rate
PF: Pulmonary fibrosis
IPF: Idiopathic pulmonary fibrosis
NPSLE: Neuropsychiatric systemic lupus erythematosus
SLE: Systemic lupus erythematosus
cIMT: Carotid intima-media thickness
cfDNA: Cell-free DNA
